# Introduction to the forensic research via omics markers in environmental health vulnerable areas (FROM) study

**DOI:** 10.4178/epih.e2024062

**Published:** 2024-07-12

**Authors:** Jung-Yeon Kwon, Woo Jin Kim, Yong Min Cho, Byoung-Gwon Kim, Seungho Lee, Jee Hyun Rho, Sang-Yong Eom, Dahee Han, Kyung-Hwa Choi, Jang-Hee Lee, Jeeyoung Kim, Sungho Won, Hee-Gyoo Kang, Sora Mun, Hyun Ju Yoo, Jung-Woong Kim, Kwan Lee, Won-Ju Park, Seongchul Hong, Young-Seoub Hong

**Affiliations:** 1 Department of Preventive Medicine, Dong-A University College of Medicine, Busan, Korea; 2 Department of Internal Medicine and Environmental Health Center, Kangwon National University, Chuncheon, Korea; 3 Department of Nano, Chemical & Biological Engineering, Seokyeong University, Seoul, Korea; 4 Environmental Health Center for Busan, Dong-A University, Busan, Korea; 5 Department of Preventive Medicine, Chungbuk National University College of Medicine, Cheongju, Korea; 6 Department of Preventive Medicine, Dankook University College of Medicine, Cheonan, Korea; 7 SD Medical Research Institute, Yongin, Korea; 8 Department of Public Health Sciences, Seoul National University, Seoul, Korea; 9 Department of Biomedical Laboratory Science, Eulji University College of Health Sciences, Seongnam, Korea; 10 Department of Convergence Medicine, Department of Digital Medicine, Asan Institute for Life Sciences, Asan Medical Center, University of Ulsan College of Medicine, Seoul, Korea; 11 Department of Life Sciences, Chung-Ang University, Seoul, Korea; 12 Department of Preventive Medicine, Dongguk University College of Medicine, Gyeongju, Korea; 13 Department of Occupational and Environmental Medicine, Chonnam National University Medical School and Chonnam National University Hwasun Hospital, Hwasun, Korea; 14 Department of Preventive Medicine, Jeju National University College of Medicine, Jeju, Korea

**Keywords:** Environment, Multiomics, Biomarkers

## Abstract

This research group (forensic research via omics markers in environmental health vulnerable areas: FROM) aimed to develop biomarkers for exposure to environmental hazards and diseases, assess environmental diseases, and apply and verify these biomarkers in environmentally vulnerable areas. Environmentally vulnerable areas—including refineries, abandoned metal mines, coal-fired power plants, waste incinerators, cement factories, and areas with high exposure to particulate matter—along with control areas, were selected for epidemiological investigations. A total of 1,157 adults, who had resided in these areas for over 10 years, were recruited between June 2021 and September 2023. Personal characteristics of the study participants were gathered through a survey. Biological samples, specifically blood and urine, were collected during the field investigations, separated under refrigerated conditions, and then transported to the laboratory for biomarker analysis. Analyses of heavy metals, environmental hazards, and adducts were conducted on these blood and urine samples. Additionally, omics analyses of epigenomes, proteomes, and metabolomes were performed using the blood samples. The biomarkers identified in this study will be utilized to assess the risk of environmental disease occurrence and to evaluate the impact on the health of residents in environmentally vulnerable areas, following the validation of diagnostic accuracy for these diseases.

## INTRODUCTION

In Korea, various sources such as industrial complexes, abandoned metal mines, and coal-fired power plants located near residential areas contribute to environmental pollution [1,2]. These facilities release a range of chemicals, contaminating the soil, water, and air with pollutants like heavy metals, which may pose significant health risks to local residents [3-6]. Consequently, health impact assessments are carried out to evaluate the extent of pollution exposure in vulnerable regions and its effects on the health of local residents [7-9]. Exposure biomarkers are used to determine the level of residents’ exposure to environmental pollutants by measuring the concentration of these substances in the body. This enables a more accurate assessment of exposure. However, the short biological half-life of these chemicals makes it challenging to confirm past exposures, as the concentrations only reflect recent exposure levels [10,11]. Previous environmental epidemiological studies have primarily been short-term, making it difficult to determine the impacts of long-term exposure to harmful environmental substances on health [12,13]. Additionally, accurate assessment of exposure is difficult, because exposure would have already stopped or worsened at the time of investigation [12,13]. In addition, as it is difficult to utilize biomarkers specific to pollutants, it is necessary to observe the actions in the body at each stage after exposure to identify the effects on health by exposure to environmental hazards. However, standardized technology is insufficient, and information on biomarkers at each stage is lacking.

Given that environmental diseases involve many complex factors and non-environmental factors, it is not easy to identify causal relationships between diseases and causative agents [14]. In addition, the identification of environmental causes of diseases is hindered by the technical challenges in defining and accurately measuring exposure, as well as dealing with variations in time, space, and among individuals [15]. Utilizing biomarkers that reflect long-term exposure can enhance the accuracy of determining past exposure to harmful substances, particularly in populations vulnerable to environmental pollution [13]. Therefore, accurate assessments of environmental exposure and risk, based on long-term exposure indicators, are crucial. To elucidate the relationship between environmental exposure and its health effects on residents of environmentally vulnerable areas, it is imperative to develop technologies usable for health impact assessments [16]. The omics technique, which provides molecular-based exposure information, facilitates more comprehensive risk assessments and can evaluate multiple exposures to various environmental hazards simultaneously [17]. Multi-omics research, which has attracted attention in recent years, is suitable for investigating causal relationships and associations between environmental exposure and causes of diseases. This is because it evaluates multiple aspects sequentially and can provide evidence for the causality of environmental hazards and environmental diseases [18]. Numerous international studies have examined the health impacts of exposure to environmentally detrimental substances, such as heavy metals and organic compounds, using multi-omics technology [19-22]. These studies have often integrated 2 or more omics datasets to identify metabolic pathways affected by exposure to environmental pollutants or to derive biomarkers associated with toxicity. However, animal models have predominated in previous biomarker research using multi-omics technology, whereas few studies have employed human biological samples.

This study has 2 primary objectives. The first is to develop multi-omics-based biomarkers that are related to exposure to environmental hazards and diseases. These biomarkers will be derived from biological samples collected through epidemiological investigations in areas that are environmentally vulnerable. The second objective is to utilize these biomarkers to assess the health impacts on residents of these areas. Through this research, we will be able to elucidate the mechanisms by which exposure to environmental hazards affects health and establish a link between the occurrence of environmental diseases and environmental exposure. By applying these biomarkers in environmental health assessments and health impact assessments for residents in environmentally vulnerable areas, we will be able to monitor environmental diseases.

## DATA RESOURCES

### Research system

**Figure 1.**
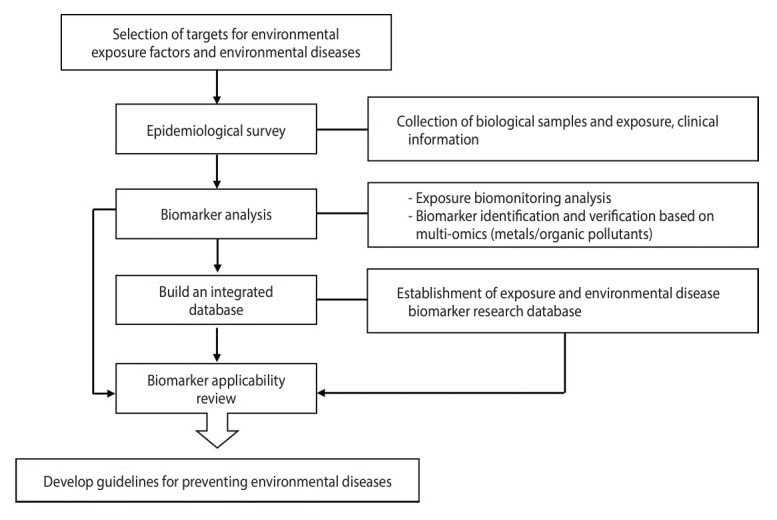
Forensic research via omics markers in environmental health vulnerable areas (FROM) study design.

The research system utilized by the study group is depicted in Figure 1. After securing biological samples in the field and developing biomarkers through the obtained samples, a system was established to manage environmentally vulnerable areas and provide a public service model by building a database.

The survey areas were selected based on major environmental hazards and diseases, with a detailed list provided in Table 1. The focus was on environmental diseases such as kidney diseases, respiratory diseases, asthma, and cancer, chosen through a review of the literature. Biological samples, including urine and blood, were collected from long-term residents in both environmentally vulnerable and control areas. Additionally, epidemiological information was gathered through surveys. These samples were assessed for exposure to environmental hazards, including heavy metals, volatile organic compounds (VOCs), and polycyclic aromatic hydrocarbons (PAHs), and analyzed for various biomarkers such as adducts, proteomes, metabolomes, and epigenomes. We intend to develop disease-specific biomarkers based on multi-omics through the analysis of individually assessed biomarkers. The identified biomarkers will be evaluated for their practical applicability, and we plan to establish a comprehensive database to support research on environmental hazard exposure and biomarkers of environmental diseases. Furthermore, this database will be utilized to develop guidelines for the diagnosis and management of environmental diseases.

### Study areas and participants

To identify environmental diseases linked to exposure in areas vulnerable to environmental health hazards, we initially identified all regions in Korea susceptible to such concerns for the selection of our study areas. Following the implementation of the Environmental Health Act and the Comprehensive Plan for Environmental Health, Korea has categorized and managed these environmentally vulnerable areas. This approach underscores the emphasized importance of preparing health protection measures for these regions.

**Figure 2.**
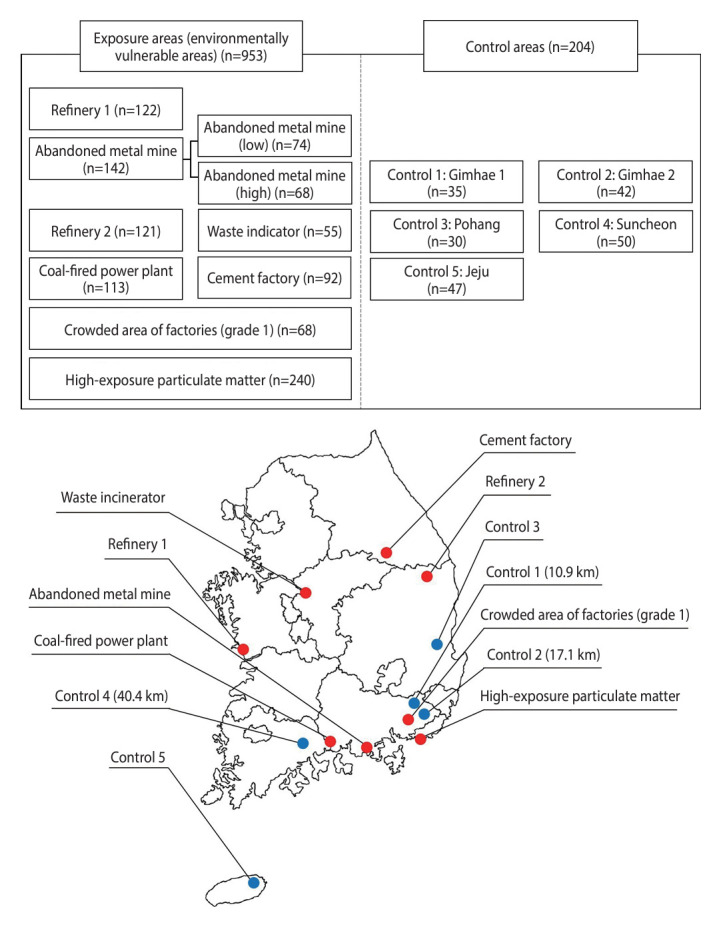
Study areas and targeted sampling sizes.

The study areas include both areas that are vulnerable in terms of environmental health and control areas. Vulnerable areas for environmental health are defined as residential zones within a 10 km radius of facilities that pose environmental health risks, potentially impacting nearby residents. This study focuses on heavy metals and organic chemicals as the primary environmental hazards. To select the study area, we analyzed reports on environmentally vulnerable areas conducted domestically from 1997 to 2021. We prioritized regions where exposure assessments for prevalent environmental hazard factors had been extensively carried out, concentrating on areas with the highest frequency of investigations [16]. The selected areas with exposure to heavy metal include those exposed refineries, and abandoned metal mining sites. Additionally, areas exposed to organic compounds, such as garbage incineration plants, thermal power plants, cement factories, industrial complexes, and regions with high exposure to particulate matter (PM), were chosen. Among the surveyed areas, the city containing the area with high exposure to PM includes ports and industrial complexes, making it particularly susceptible to air pollution from ship and factory emissions. According to a previous study [23], this area exhibits higher concentrations of PM (PM_10_ and PM_2.5_) than other city regions. Furthermore, recent data (2018-2022) from Air Korea’s Real-time Air Quality Monitoring System indicated a high average concentration of PM_10_, leading to the selection of this area for the survey. Control areas were selected based on their location—at least 10 km away from any factories or environmentally vulnerable facilities—and their demographic similarities (age groups, gender ratios) to the exposure areas. In total, 13 areas were investigated: 8 exposure areas and 5 control areas. The exposure areas included 1 in Gangwon Province (cement factory), 1 in Gyeongsangbuk Province (refinery), 4 in Gyeongsangnam Province (abandoned metal mine, coal-fired power plant, crowded area of factories: grade 1), and area with high exposure to particulate matter, and 2 in Chungcheong Province (refinery and waste incineration plant). The control areas comprised 2 in Gyeongsangnam Province, 1 in Gyeongsangbuk Province, 1 in Jeollanam Province, and 1 in Jeju Province. The locations of these areas are depicted in Figure 2; the red dots represent the exposure areas, and the blue dots represent the control areas. Control areas 1, 2, and 4, shown nearby on the map, indicate the straight-line distances to the adjacent exposed areas (Figure 2).

In total, 1,157 participants were recruited from areas vulnerable to environmental health risks and from control areas. Recruitment occurred between June 2021 and October 2022 for participants from regions exposed to heavy metals, and between May 2023 and September 2023 for those from regions exposed to organic compounds, along with additional control areas. The recruitment period spanned 2 years and 4 months. All participants were adults aged 19 or older who had resided in their respective areas for at least 10 years. To engage all residents from the targeted areas, the objectives and methods of the study were thoroughly explained. The survey was then conducted among those who voluntarily agreed to participate. Figure 2 includes a schematic diagram that illustrates the distribution of participants across the survey regions. The surveys collected basic demographic data and health-related information, including smoking history, alcohol consumption, and disease history.

### Ethics statement

This study was conducted after review and approval of the study protocol by the Institutional Review Board of Dong-A University (IRB Nos. 2021: 2-1040709-AB-N-01-202105-BR-002-08, 2022: 2-1040709-AB-N-01-202105-BR-002-12, 2023: 2-1040709-AB-N-01-202105-BR-002-16). The participants provided written informed consent.

### Biological sample collection

The blood and urine samples collected from participants were delivered to various laboratories for analysis of environmental hazardous factors and omics. The samples were distributed as follows: Dong-A University received samples for heavy metal analysis; SD Medical Research Institute handled VOCs and PAHs; Chungbuk National University focused on adducts; Chung-Ang University and Kangwon National University both conducted epigenome studies; Asan Medical Center in Seoul analyzed the metabolome; and Eulji University examined the proteome.

**Figure 3.**
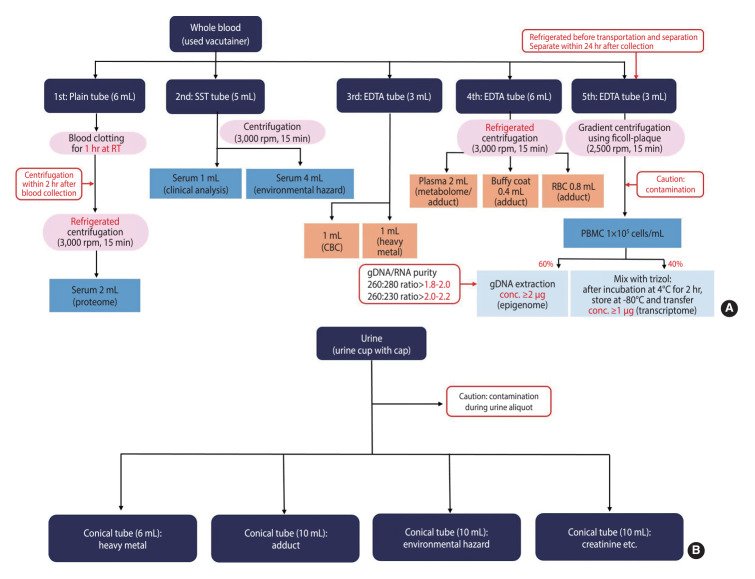
Biological sample collection procedure (A) blood, (B) urine. SST, serum separation tube; EDTA, ethylene diamine tetraacetic acid; RT, room temperature; RBC, red blood cell; CBC, complete blood count; PBMC, peripheral blood mononuclear cells; conc., concentration.

Three types of blood collection tubes were utilized for blood collection. Venous blood was collected using a plain tube without any anticoagulant, a serum separation tube (SST), and a tube containing ethylene diamine tetraacetic acid (EDTA) as an anticoagulant. The blood in the plain tube was centrifuged at 3,000 rpm for 15 minutes under refrigerated conditions within 2 hours of collection. The blood in the SST tube was allowed to sit at room temperature for 30 minutes before the supernatant was separated by centrifugation at 3,000 rpm for 15 minutes. The blood in the EDTA tube was agitated by shaking 8-10 times to prevent clotting and then roll mixed on an agitator for 30 minutes. Samples for adduct analysis from the EDTA tubes were centrifuged at 3,000 rpm for 15 minutes in a refrigerated environment to separate the plasma, buffy coat, and red blood cells. Additionally, blood for DNA and RNA isolation was pre-treated within 24 hours of collection. The separated components were transported under refrigeration and stored frozen until analyzed. Spot urine was collected using a spot urine cup, transferred into a 15 mL tube, and then transported under refrigeration to each analysis institution. The procedures for collecting and separating blood and urine samples by exposure assessment item are illustrated in Figure 3.

### Exposure assessment

Various types of biomarkers are analyzed using the obtained biological samples to assess exposure to environmental hazards. The environmental hazards and biomarkers analyzed in this study, along with the biological samples used, are listed in Table 2. The analysis methods for each item are described below.

#### Heavy metals

An analysis of heavy metals in blood and urine is conducted using various methods: an inductively coupled plasma mass spectrometer (ICP-MS) for lead, cadmium, and total arsenic; an automated mercury analyzer for mercury; and high-performance liquid chromatography with inductively coupled plasma mass spectrometry (HPLC-ICP-MS) for arsenic speciation. Blood and urine samples for heavy metal analysis are taken at room temperature and thoroughly mixed using a roll mixer for at least 30 minutes. The standard solution of heavy metals to be analyzed is progressively diluted and used. To ensure the stability of the analysis, a specific concentration from the calibration curve and the standard substance are analyzed after every 20 samples to verify the calibration curve. Heavy metals analyzed via ICP-MS are assessed by diluting blood and urine samples by 1/10 or 1/40, depending on the analysis equipment. Blood mercury analysis is performed by directly injecting the blood into the sample container of the automatic mercury analyzer. The separation of urine for arsenic species using HPLC-ICP-MS involves filtering urine samples and diluting them 1/10 in a mobile phase before analysis.

#### Adducts

DNA adduct analysis was conducted in two phases: the non-targeted and targeted methods. Candidate adducts were initially identified through non-targeted analysis using blood and urine samples. For the analysis of blood DNA adducts, genomic DNA was extracted from the buffy coat, and 20 μg of DNA was subsequently digested and purified using DNase I, phosphodiesterase I, and alkaline phosphatase. Urinary DNA adducts were analyzed after purifying 1 mL of urine via solid-phase extraction. Non-target DNA adduct analysis utilized high-resolution MS (Q-TOF) CNL scan, which helped confirm an exposure-specific adduct profile with significant differentiation between the exposed and control groups. This confirmed profile was then selected as the final candidate DNA adduct using the DNA adduct database [24]. For the analysis of targeted urinary DNA adducts, an internal standard was added to the urine sample, which was then purified and extracted using solid phase extraction. The analysis was performed using LC-QqQ-MS/MS (triple quadrupole mass spectrometry) in multiple-reaction monitoring (MRM) mode.

#### Proteome

In the study of proteomes, serum is first separated from blood, and the highly abundant serum proteins are removed using a multiple-affinity removal system. Subsequently, 100 μg of protein was prepared for reduction using 5 mM Tris (2-carboxyethyl) phosphine (Pierce, Rockford, IL, USA), alkylation with 15 mM iodoacetamide (Sigma-Aldrich, St. Louis, MO, USA), and peptide digestion by MS-grade trypsin gold (Promega, Madison, WI, USA). The resulting peptides were then desalted using a C18 cartridge from Waters (Milford, MA, USA). Following desalting, the samples were loaded onto an Eksigent ChromXP nanoLC column (75 µm i.d.× 15 cm) for separation and subsequently analyzed by sequential windowed acquisition of all theoretical fragment ion mass spectra (SWATH) acquisition using a TripleTOF 5600 mass spectrometer (AB Sciex, Concord, ON, Canada). The extraction of peaks from the raw data generated by SWATH acquisition and protein quantitation was carried out using PeakView software.

#### Metabolome

Metabolites were extracted from plasma samples using liquid-liquid extraction (LEE) [25,26]. For global metabolome profiling, we employed liquid chromatography-mass spectrometry (LCMS/MS) with a Dionex Ultimate3000 and LTQ-Orbitrap XL system (Thermo Fisher Scientific, Waltham, MA, USA), operating in both positive and negative ion modes. Pooled human plasma served as the quality control (QC) standard, with samples evenly distributed across all batches. Each batch included two QC samples, and the order of analysis was randomized within each batch. Metabolomic features (m/z and retention time) were extracted using Compound Discoverer 3.3 (Thermo Fisher Scientific). Metabolites were identified through database searches based on accurate masses within a 10 ppm mass tolerance, isotopic abundance, and MS2 library matching.

Amino acids, fatty acids, sphingolipids, phospholipids, and other polar metabolites, including those related to energy metabolism, were quantified using targeted metabolomics platforms. The majority of metabolites analyzed were extracted using LLE, with the exception of free fatty acids, which were extracted using isooctane. Amino and free fatty acids underwent chemical derivatization using phenylisothiocyanate and BCl3-methanol, respectively. Internal standards were added to calibration solutions, sample plasmas, and QCs prior to sample preparation. Metabolites were identified using liquid chromatography-tandem mass spectrometry (LCMS/MS, Agilent1290-Qtrap5500) and gas chromatography-mass spectrometry (GC-MS, Agilent 7890/5975). Detailed procedures for detecting sphingolipids, free fatty acids, amino acids, and phospholipids are available in recent publications [27-30]. MRM was used in negative ion mode for data analysis. Data analysis for all metabolites, except free fatty acids, was conducted using Analyst 1.5.2 (ABSciex), while MSD Chemstation software (Agilent E02.02.1431) was utilized for the analysis of free fatty acids.

#### Epigenome

Reduced representation bisulfite sequencing libraries were constructed using genomic DNA isolated from human peripheral blood mononuclear cells. The isolated genomic DNA was digested with MspI and ligated to two adapters: adapter-A, which provides a binding site for the Illumina R1 sequencing primer, and adapter-B, which provides a binding site for the Illumina R2 sequencing primer. Following bisulfite conversion was performed, and, the DNA underwent two rounds of polymerase chain reaction amplification to produce sequencing-ready libraries. The quality and quantity of the libraries were assessed using a Bioanalyzer, and the libraries were subsequently sequenced on an Illumina Hiseq platform.

#### Organic compounds

The study analyzed various organic compounds as exposure markers, including four metabolites of VOCs (phenylglyoxylic acid, trans, trans-muconic acid, methylhippuric acid, and benzyl-mercapturic acid) found in urine. Additionally, four metabolites of PAHs (2-naphthol, 1-hydroxypyrene, 2-hydroxyfluorene, and 1-hydrophenanthrene) were detected in urine, along with a metabolite of environmental tobacco smoke (cotinine) also in urine. The study further examined 25 persistent organic pollutants in serum, including beta-HCH, gamma-HCH, HCB, p,p’-DDE, p,p’-DDT, 4CB-52, 4CB-56, 4CB-74, 5CB-101, 5CB-105, 5CB-118, 5CB-123, 5CB-126, 6CB-138, 6CB-153, 6CB-157, 6CB-167, 7CB-180, 7CB-187, 3BDE-28, 4BDE-47, 5BDE-99, 5BDE-100, 6BDE-153, and 6BDE-154. Additionally, 5 perfluorinated compounds were analyzed in serum: perfluorooctanoic acid, perfluorooctane sulfonic acid, perfluorohexane sulfonic acid, perfluorodecanoic acid, and perfluorononanoic acid.

Metabolites of VOCs and cotinine in urine samples were analyzed using LC-MS/MS, while metabolites of PAHs were analyzed using GC-MS. Persistent organic compounds in serum were analyzed using GC-HRMS, and perfluorinated compounds were analyzed using LC-MS/MS. All steps of the analysis, including sample preparation, chemical analysis, internal and external QC, and reporting of results, adhered to the protocols of the Korean National Environmental Health Survey [31,32].

#### Oxidative damage biomarkers

The concentration of urinary 8-OHdG was determined using a highly sensitive 8-hydroxy-2´-deoxyguanosine enzyme-linked immunosorbent assay kit (JalCA, Shizuoka, Japan), following the manufacturer’s instructions. Urinary malondialdehyde levels were measured using HPLC with a fluorescence detector, based on a 2-thiobarbituric acid assay.

#### Kidney function indicators

Kidney function indicators are analyzed using urine samples. N-acetyl-β-glucosaminidase (NAG) is analyzed through a colorimetric method, using the NAG test kit (Nittobo Medical, Tokyo, Japan). β2-microglobulin (β2-MG) is analyzed via an immuno-turbidimetric assay using the β2-MG test kit (Roche, Basel, Switzerland). Creatinine is analyzed using the modified Jaffe method using kits from Roche.

## DATA RESOURCE USE

### Socio-demographic characteristics of the study participants

The main demographic characteristics of the participants are presented in Table 3, which includes data from both exposed and control areas. The participant group comprised 35.5% men and 64.5% women. There was no significant difference in the gender distribution between the exposed and control areas. The average age was 70.5 years in the exposed area and 68.7 years in the control areas. In the exposed areas, 37.0% of participants were in their 70s, while in the control areas, 37.7% were in their 60s, representing the largest age category in each group. The average duration of residence was 36 years for both areas. Regarding current occupations, 39.2% of participants were unemployed, likely due to the older average age. Agriculture and fishing were the next most common occupations, comprising 27.7% of the workforce. A majority of participants (71.2%) were non-smokers. The proportions of current drinkers and non-drinkers were similar across both the exposed and control areas. The primary source of drinking water for 61.5% of participants was either purified or bottled water. Regarding dietary habits, 77.2% of participants consumed fish and 73.1% consumed seafood less than three times a week. On average, over 20% of participants in both exposed and control areas were self-sufficient (accounting for over half of production) in rice, vegetables, potatoes, and seeds, with the control areas exhibiting higher self-sufficiency rates than the exposed areas.

### Disease history of study participants

Table 4 shows the disease history of the study participants as diagnosed in hospitals. Among the chronic diseases, hypertension had a higher diagnosis rate in the exposed area at 49.5%, compared to 39.7% in the control area. The diagnosis rate of diabetes was 20.7% in the exposed area and 19.6% in the control area, with no statistically significant difference between the two. Although the rates for lung disease, asthma, and kidney disease were relatively low, they were more than twice as high in the exposed area than in the control area. Notably, there were no cases of respiratory-related cancers (lung, bronchial, and laryngeal cancers) diagnosed in the control areas, whereas these cancers were diagnosed in 1.4% of participants in the exposed areas.

Two studies using the obtained epidemiological information and biological specimens have been recently published [3,33]. The analysis focused on participants from selected regions, rather than the entire cohort. In this study, we observed a difference in the level of exposure to heavy metals between the exposure areas and the control areas, which correlated with the kidney functional indicator.

Herein, we aim to introduce the purpose and system of the forensic research via omics markers in environmental health vulnerable areas (FROM) study, as well as present research findings, including the demographic characteristics of study participants obtained through epidemiological investigation. We are currently conducting extensive biomarker analysis on all participants; therefore, the results are not yet available. Once data analysis is complete, we will publish a follow-up paper detailing the results of the biomarker analysis.

## STRENGTHS AND WEAKNESSES

This study provides an overview of the development of biomarkers affected by long-term exposure to environmental hazards among residents of areas vulnerable to environmental health risks. To explore the potential health effects of environmental hazards, we gathered epidemiological data concerning lifestyle and disease history. Additionally, we collected ample biological samples to facilitate the development of biomarkers and assess exposure to environmental hazards.

The differentiation and advantages of the FROM study group’s research on environmentally vulnerable areas are as follows. First, this study was conducted in conjunction with field epidemiology and experimental research. We were able to obtain samples from residents in environmentally vulnerable areas by securing biological samples on-site. These samples were then transported to analysis institutions for exposure assessment of environmental hazards and biomarker analysis, ensuring stable sample collection and transportation for biomarker development. Second, this study investigated more than five diverse environmentally vulnerable areas, each with different exposure characteristics, rather than assessing exposure in areas with uniform characteristics. The study areas included industrial complexes, abandoned metal mining areas, high-exposure particulate matter areas, and waste treatment facilities (incineration plants). Additionally, areas where diseases had already occurred or were a concern due to environmental hazards, such as coal-fired power plant areas, refinery areas, and cement factory areas, were also investigated. This study not only investigated various environmentally vulnerable areas but also conducted surveys in control areas to evaluate differences between environmentally vulnerable areas and control areas, as well as within the vulnerable areas themselves. Through this analysis, it will be possible to identify differentiated biomarkers according to the exposure characteristics of environmentally vulnerable areas or areas of high exposure to certain environmental hazards. Third, we obtained sufficient biological samples for biomarker analysis. It is essential to collect an adequate number of biological samples to elucidate the biological mechanisms of diseases caused by exposure to environmental hazards. To this end, a pilot study was conducted on a small number of participants to determine the number of biological samples sufficient for biomarker analysis. Additionally, errors in the preparation process were examined to establish a biological sample collection system. Based on this, we obtained sufficient biological samples for biomarker analysis. However, as we did not collect biological samples such as tissue or hair, it was impossible to examine all environmental exposures or conduct genetic studies. Nevertheless, it is believed that the biological samples currently obtained are sufficient for research on biomarkers caused by environmental exposure. Furthermore, the biological sample collection system established in this study is expected to be useful in other studies for biomarker analysis.

This study has a few limitations. First, over 80% of the participants were aged 60 years or older, predominantly categorizing them as older adults. Consequently, their disease history and the levels of environmental toxins in their bodies might have been affected not only by exposure to environmentally vulnerable facilities but also by their age. Thus, it is crucial to account for the influence of age when evaluating exposure to environmental hazards and analyzing biomarkers. Second, the study employed a cross-sectional design instead of a cohort-based follow-up study. This approach was chosen because the study’s objective was to develop biomarkers for areas particularly susceptible to environmental health risks. Cross-sectional research was conducted in areas where the impact of environmentally vulnerable facilities was evident. To validate biomarkers specific to each environmentally sensitive area, further studies are necessary in different vulnerable regions that share characteristics with the areas previously examined, rather than conducting follow-up studies in the same locations.

In conclusion, ongoing research is essential in areas where environmentally vulnerable facilities contribute to environmental and health damage. It is crucial to improve the current health impact assessments, which have been limited to exposure assessments. Utilizing biomarkers makes it possible to identify the causes of environmental diseases linked to these vulnerable areas and elucidate the relationships between these causes and the resultant diseases. Consequently, the biomarkers developed in this study are expected to be applicable to future health impact assessments in areas vulnerable to environmental health concerns.

## Figures and Tables

**Table 1.** Major areas vulnerable in terms of environmental healths in Korea

Pollution source	Environmental hazards	Environmental disease (expected)
Abandoned metal mines	Heavy metals, etc.	Kidney disease, cancer, osteoporosis, respiratory diseases, etc.
Refineries (smelters)	Heavy metals, gaseous substance, etc.	
Industrial complexes	Heavy metals, VOCs, PAHs, PMs (fine dust), gaseous substances, phthalates, etc.	Asthma, chronic obstructive pulmonary disease (respiratory diseases), allergic disease, etc.
Waste incinerators	Heavy metals, PM, POPs, VOCs, etc.	
Coal-fired power plants	PMs, heavy metals, PAHs, gaseous substance, electromagnetic waves, etc.	
Cement factories	Heavy metals, VOCs, PAHs, PM, etc.	

VOCs, volatile organic compounds; PAHs, polycyclic aromatic hydrocarbons; PM, particulate matter; POPs, persistence organic pollutants.

**Table 2.** Analysis items for environmental hazard factors in different biological samples

Biological sample	Environmental hazards
Blood	Heavy metals (Pb, Cd, Hg); Adducts; Proteomes; Metabolomes; Epigenomes; POPs (beta-HCH, gamma-HCH, HCB, p,p’-DDE, p,p’-DDT, 4CB-52, 4CB-56, 4CB-74, 5CB-101, 5CB-105, 5CB-118, 5CB-123, 5CB-126, 6CB-138, 6CB-153, 6CB-157, 6CB-167, 7CB-180, 7CB-187, 3BDE-28, 4BDE-47, 5BDE-99, 5BDE-100, 6BDE-153, and 6BDE-154); PFCs (perfluorooctanoic acid, perfluorooctane sulfonic acid, perfluorohexane sulfonic acid, perfluorodecanoic acid and perfluorononanoic acid)
Urine	Heavy metals (Cd, As, As speciation); Adducts; VOCs (PGA, tt-MA, MHA, BMA); PAHs (2-naphthol, 1-Hydroxypyrene, 2-Hydroxyfluorene, 1-Hydroxyphenanthrene); Nicotinine metabolites; Oxidative damage biomarkers (8-OHdG, MDA); Kidney function indicators (β2-MG, NAG)

Pb, lead; Cd, cadmium; Hg, mercury; POPs, persistence organic pollutants; beta-HCH, beta-Hexachlorocyclohexane; gamma-HCH, gamma-hexachlorocyclohexane; HCB, hexachlorobenzene; p,p’-DDE, p,p’-dichlorodiphenyldichloroethylene; p,p’-DDT, p,p’-dichlorodiphenyltrichloroethane; 4CB-52, 2,2’,5,5’-tetrachlorobiphenyl; 4CB-56, 2,3,3’,4’-tetrachlorobiphenyl; 4CB-74, 2,4,4’,5-tetrachlorobiphenyl; 5CB-101, 2,2’,4,5,5’-pentachlorobiphenyl; 5CB-105, 2,3,3’,4,4’-pentachlorobiphenyl; 5CB-118, 2,3’,4,4’,5-pentachlorobiphenyl; 5CB-123, 2,3’,4,4’,5’-pentachlorobiphenyl; 5CB-126, 3,3’,4,4’,5-pentachlorobiphenyl; 6CB-138, 2,2’,3,4,4’,5’-hexachlorobiphenyl; 6CB-153, 2,2’,4,4’,5,5’-hexachlorobiphenyl; 6CB-157, 2,3,3’,4,4’,5’-hexachlorobiphenyl; 6CB-167, 2,3’,4,4’,5,5’-hexachlorobiphenyl; 7CB-180, 2,2’,3,4,4’,5,5’-heptachlorobiphenyl; 7CB-187, 2,2’,3,4’,5,5’,6-heptachlorobiphenyl; 3BDE-28, 2,4,4’-tribromodiphenyl ether; 4BDE-47, 2,2’,4,4’-tetrabromodiphenyl ether; 5BDE-99, 2,2’,4,4’,5-pentabromodiphenyl ether; 5BDE-100, 2,2’,4,4’,6-pentabromodiphenyl ether; 6BDE-153, 2,2’,4,4’,5,5’-hexabromodiphenyl ether; 6BDE-154, 2,2’,4,4’,5,6’-hexabromodiphenyl ether; PFCs, perfluorinated compounds; As, arsenic; VOCs, volatile organic compounds; PGA, phenylglyoxylic acid; tt-MA, trans, trans-muconic acid; MHA, methylhippuric acid; BMA, benzyl-mercapturic acid; PAHs, polycyclic aromatic hydrocarbons; 8-OHdG, 8-hydroxy-2’-deoxyguanosine; MDA, Malondialdehyde; b2-MG, beta 2-microglobulin; NAG, N-acetyl-beta-D-glucosaminidase.

**Table 3.** General characteristics of the study participants

Characteristics			Total	Exposure area	Control area	p-value
Total			1,157 (100)	953 (82.4)	204 (17.6)	
Gender		Men	411 (35.5)	334 (35.0)	77 (37.7)	0.465^2^
		Women	746 (64.5)	619 (65.0)	127 (62.3)	
Age (yr)		Mean±SD	70.2±10.2	70.5±10.2	68.7±9.8	0.020^1^
		≤59	151 (13.1)	118 (12.4)	33 (16.2)	0.055^2^
		60-69	374 (32.3)	297 (31.2)	77 (37.7)	
		70-79	417 (36.0)	353 (37.0)	64 (31.4)	
		≥80	215 (18.6)	185 (19.4)	30 (14.7)	
Residence period (yr)		Mean±SD	36.0±22.4	35.8±22.3	36.9±22.7	0.532^1^
		≤20	385 (33.3)	322 (33.8)	63 (30.9)	0.707^2^
		21-40	303 (26.2)	252 (26.4)	51 (25.0)	
		41-60	281 (24.3)	228 (23.9)	53 (26.0)	
		≥61	188 (16.2)	151 (15.8)	37 (18.1)	
Current job		Agriculture & fishery	321 (27.7)	214 (22.5)	107 (52.5)	<0.001^2^
		Service work	84 (7.3)	73 (7.7)	11 (5.4)	
		Simple labor	143 (12.4)	134 (14.1)	9 (4.4)	
		Unemployed	453 (39.2)	387 (40.6)	66 (32.4)	
		Other	156 (13.5)	145 (15.2)	11 (5.4)	
Monthly income (10,000 Korean won)		Unknown	51 (4.4)	42 (4.4)	9 (4.4)	0.309^2^
		Low (<50)	357 (30.9)	297 (31.2)	60 (29.4)	
		Medium (≥50, <200)	462 (39.9)	388 (40.7)	74 (36.3)	
		High (≥200)	287 (24.8)	226 (23.7)	61 (29.9)	
Smoking status		Current	94 (8.1)	75 (7.9)	19 (9.3)	0.710^2^
		Past	239 (20.7)	195 (20.5)	44 (21.6)	
		Never	824 (71.2)	683 (71.7)	141 (69.1)	
Drinking (alcohol) status		Current	464 (40.1)	378 (39.7)	86 (42.2)	0.059^2^
		Past	259 (22.4)	226 (23.7)	33 (16.2)	
		Never	434 (37.5)	349 (36.6)	85 (41.7)	
Drinking water		Tap water	390 (33.7)	327 (34.3)	63 (30.9)	0.003^2^
		Purified water	712 (61.5)	590 (61.9)	122 (59.8)	
		Ground water	55 (4.8)	36 (3.8)	19 (9.3)	
Fish intake frequency (usually)		None	165 (14.3)	133 (14.0)	32 (15.7)	0.792^2^
		≤3 times/wk	893 (77.2)	739 (77.5)	154 (75.5)	
		≥4 times/wk	99 (8.6)	81 (8.5)	18 (8.8)	
		No response	0 (0.0)	0 (0.0)	0 (0.0)	
Seafood intake frequency (usually)		None	155 (13.4)	119 (12.5)	36 (17.6)	0.006^3^
		≤3 times/wk	846 (73.1)	692 (72.6)	154 (75.5)	
		≥4 times/wk	151 (13.1)	137 (14.4)	14 (6.9)	
		No response	5 (0.4)	5 (0.5)	0 (0.0)	
Seafood intake within the last 1 day		Yes	515 (44.5)	421 (44.2)	94 (46.1)	0.034^3^
		No	640 (55.3)	532 (55.8)	108 (52.9)	
		No response	2 (0.2)	0 (0.0)	2 (1.0)	
Local food self-sufficiency (%)	Rice	≥50	256 (22.1)	199 (20.9)	57 (27.9)	0.027^2^
		<50	901 (77.9)	754 (79.1)	147 (72.1)	
	Vegetables	≥50	528 (45.6)	400 (42.0)	128 (62.7)	<0.001^2^
		<50	629 (54.4)	553 (58.0)	76 (37.3)	
	(Sweet and) Potatoes	≥50	304 (26.3)	211 (22.1)	93 (45.6)	<0.001^2^
		<50	853 (73.7)	742 (77.9)	111 (54.4)	
	Nuts and seeds	≥50	270 (23.3)	199(20.9)	71(34.8)	<0.001^2^
		<50	887 (76.7)	754 (79.1)	133 (65.2)	

Values are presented as number (%). SD, standard deviation.

1 Using the Student t-test.

2 Using the chi-square test.

3 Using the Fisher’s exact test.

**Table 4.** Health effects (medical history) of the study participants

Variables	Total	Exposure area	Control area	p-value^1^
Total	1,157 (100)	953 (82.4)	204 (17.6)	
Hypertension				0.011
Yes	553 (47.8)	472 (49.5)	81 (39.7)	
No	604 (52.2)	481 (50.5)	123 (60.3)	
Diabetes				0.733
Yes	237 (20.5)	197 (20.7)	40 (19.6)	
No	920 (79.5)	756 (79.3)	164 (80.4)	
Chronic obstructive pulmonary disease				0.019
Yes	53 (4.6)	50 (5.2)	3 (1.5)	
No	1,104 (95.4)	903 (94.8)	201 (98.5)	
Asthma				0.049
Yes	53 (4.6)	49 (5.1)	4 (2.0)	
No	1,104 (95.4)	904 (94.9)	200 (98.0)	
Allergic disease (dermatitis/rhinitis)				0.042
Yes	77 (6.7)	70 (7.3)	7 (3.4)	
No	1,080 (93.3)	883 (92.7)	197 (96.6)	
Kidney disease				0.109
Yes	38 (3.3)	35 (3.7)	3 (1.5)	
No	1,119 (96.7)	918 (96.3)	201 (98.5)	
Arthritis				0.015
Yes	306 (26.4)	266 (27.9)	40 (19.6)	
No	851 (73.6)	687 (72.1)	164 (80.4)	
Lung/Bronchial/Laryngeal cancer				0.093
Yes	13 (1.1)	13 (1.4)	0 (0.0)	
No	1,144 (98.9)	940 (98.6)	204 (100)	
Other cancers				0.917
Yes	110 (9.5)	91 (9.5)	19 (9.3)	
No	1,047 (90.5)	862 (90.5)	185 (90.7)	

Values are presented as number (%).

1 Using the chi-square test.

## Data Availability

The datasets generated this study are not publicly available but are available from the corresponding author on reasonable request. For further information, contact Professor Young-Seoub Hong, the principal investigator (yshong@dau.ac.kr).
